# A Distinct Second Spectral Peak in White Matter BOLD Signals: Mathematical Modeling, Spatial Distribution and Age-Related Evolution

**DOI:** 10.21203/rs.3.rs-10770821/v1

**Published:** 2026-08-27

**Authors:** sijing wu, Muwei Li, Yurui Gao, Lyuan Xu, Yu Zhao, John C Gore, Zhaohua Ding

**Affiliations:** Vanderbilt University Medical Center; Vanderbilt University Medical Center; Vanderbilt University; Oregon Institute of Technology; West China Hospital of Sichuan University; Vanderbilt University Medical Center; Vanderbilt University Medical Center

## Abstract

White matter blood-oxygenation-level-dependent (BOLD) signals in resting-state functional magnetic resonance imaging (rs-fMRI) are increasingly recognized for their physiological significance, yet their intrinsic spectral architecture remains largely uncharted. In this work, using high quality data from the Human Connectome Project (HCP), we mathematically model and quantitatively characterize a robustly detectable “second peak” superimposed on the canonical exponentially decaying white matter BOLD spectrum. The spectral signature, with a peak frequency around ~ 0.06 Hz, emerges as a stable individual-level phenotype with broad spatial penetrance across white matter. Cross-tissue analyses reveal that while BOLD signals in cerebrospinal fluid (CSF) exhibit similar low frequency dynamics, white matter provides the most prominent and organized manifestation of this spectral feature. Spatially, the peak follows distinct gradients that mirror macroscopic white matter anatomy. Exploratory analyses in the HCP-Aging cohort further suggest that this band-specific spectral signature undergoes systematic evolution with age. Synthesizing the frequency profile, spatial distribution, and aging trajectory, we propose that the white matter second peak may serve as an organized, tissue-modulated readout of ubiquitous low-frequency physiological dynamics, potentially linked to glymphatic or perivascular clearance mechanisms in the brain. Together, these findings point to a robust spectral phenotype that may open a new noninvasive dimension for investigating white matter functional integrity, brain aging, and relevant physiological mechanisms.

## 1 Introduction

Brain white matter (WM) has long taken a paradoxical position in resting-state functional magnetic resonance imaging (rs-fMRI): it is anatomically indispensable, yet often functionally overlooked (Gore et al., 2019). Traditionally, blood-oxygen-level–dependent (BOLD) signals in WM were treated as a nuisance background, but accumulating evidence has now suggested that they carry detectable and physiologically meaningful functional information, motivating renewed interest in WM fMRI in its own right (Ding et al., 2018; Gawryluk et al., 2014; Huang et al., 2023; Ji et al., 2017; Peer et al., 2017). To date, prior work has predominantly focused on WM functional connectivity (FC), typically drawn upon temporal correlations between BOLD signals across WM regions (see, for instance, Li et al, 2019), which has revealed functionally relevant organizations within WM (Wang et al., 2020). The FC analyses, along with those based on summative statistical descriptions of power spectra of WM BOLD signals, have been extensively pursued lately, offering unprecedented understanding of the pathophysiology of the human brain (see Gore et al., 2026 for review).

A puzzling observation in resting-state WM BOLD signals is the saliently distinct peak around the frequency of 0.05–0.06 Hz in their power spectral profile. Our previous analysis demonstrated that this peak could relate to certain features in the BOLD signal time course (Li et al., 2021). More recently, we have unexpectedly found that a similar spectral peak also robustly exists in the BOLD signals of cerebrospinal fluid (CSF) in the lateral ventricles. Unlike the monotonic exponential decay commonly seen in gray matter (GM) BOLD signals, the superimposed spectral peak in WM and CSF, referred to as “second peak” of the power spectra henceforth, calls for a revisit of this enigmatic phenomenon.

The current work was thus motivated to analyze the second spectral peak in WM more comprehensively using large cohorts of high quality rs-fMRI data sourced from the Human Connectome Project (HCP). We examined the second peak at the group, subject, and voxel levels, characterized its spatial distributions and robustness, and further explored the evolution of this spectral feature with age in the HCP-Aging cohort. This study established a basic descriptive and quantitative modeling framework for the unusual spectral phenomenon and discussed its potential physiological significance.

## Materials and Methods

### 2.1 Datasets

This study used two datasets from the Human Connectome Project. The primary analyses were conducted using rs-fMRI data from 120 healthy young adults (22–36 years, #female = 60) from the HCP S1200 release (Van Essen et al., 2013). All participants completed the standard HCP rs-fMRI protocol (TR = 0.72 s, 2 mm isotropic voxels, approximately 15 minutes per session). The imaging data had been preprocessed with the standard HCP pipeline, including motion correction, field inhomogeneity correction, registration to MNI152 standard space, and regression of individualized motion parameters and physiological noises. The primary analyses were extended into the HCP-Aging dataset (Bookheimer et al., 2019) to include participants of a broader age range. These participants were grouped by decade, including 31–40 years ( n = 84), 41–50 years ( n = 141), 51–60 years ( n = 160), 61–70 years ( n = 114), 71–80 years ( n = 104), and 81–90 years ( n = 72). The same analysis framework was applied across the datasets and all the age groups.

### 2.2 Generation of tissue masks and preprocessing of BOLD time series

Individualized GM, WM and CSF masks were derived from SPM12 tissue probability maps using standard thresholds. The GM and WM masks were further constrained by a group-level mask, and the CSF mask was eroded to avoid overlapping with GM and WM voxels. Prior to spectral analysis, BOLD time series were spatially smoothed within each tissue mask, and then temporally detrended and z-score normalized according to standard procedures.

### 2.3 Estimation of signal power spectral density

For each voxel, power spectral density was estimated from the preprocessed BOLD time series using Welch’s method (Welch, 1967). Subsequent analyses were based on the amplitude spectrum within the frequency range of *f* = 0.01–0.20 Hz, as employed previously (Ding et al., 2025). The spectra were averaged within each tissue mask and then across participants to derive individual-level and group-level mean spectra respectively.

### 2.4 Parameterization of the second spectral peak

To characterize the second peak in the WM spectrum, the amplitude spectrum was empirically fitted over the frequency range of f=0.01−0.20Hz with a combined exponential-Lorentzian model commonly used for modeling superposition of a narrow rhythmic peak on a monotonically falling broadband background that flattens at high frequency:

(1) $$S(f)=c+ae^{-bf}+\frac{A}{\pi }\frac{\gamma }{{\left(f-f_{0}\right)}^{2}+\gamma^{2}}.$$

The observed amplitude spectrum S(f) was modeled as a Lorentzian function superimposed onto an exponentially decaying background (defined by parameters a, b, c), from which peak area $\left(A\right)$, center frequency $\left(f_{0}\right)$ and half width parameter (γ) were extracted and peak height (A/(π⋅γ)) derived. The model parameters were estimated by nonlinear least squares minimization, with model adequacy assessed by comparing against a pure exponential model using Akaike information criterion (AIC) and the coefficient of determination (R^2^). For voxelwise analyses, only stable fits with center frequency between 0.03 and 0.15 Hz, peak half width γ≤0.06Hz, peak height exceeding 5% of the estimated exponential background at $f_{0}$, and R^2^ ≥ 0.5 were retained. The spectral peak in these voxels was defined as a valid peak, and the WM voxels containing a valid peak were defined valid voxels.

### 2.5 Analysis of spatial distributions of Lorentzian parameters

Based on the voxelwise Lorentzian fitting above, spatial maps of peak area, center frequency, peak width $\left(2^{*}\;\gamma \right)$ and height in WM were generated. These maps were averaged across subjects to obtain group-level spatial distributions. The proportion of stable fit across participants was also computed voxelwise to assess the spatial stability of the parameter maps, with quality gauged by fit R^2^.

### 2.6 Evaluation of energy contributions from Lorentzian component

To quantify energy contributions from the Lorentzian component to the observed power spectral energy, we chosen a frequency band (0.03–0.09 Hz) around the center frequency $f_{0}$ of the second spectral peak and defined:

(2) $${\text{Contribution}}_{0.03-0.09}=\frac{\int_{0.03}^{0.09}\max\left[y_{\text{obs}}(f)-E(f),0\right]df}{\int_{0.03}^{0.09}y_{\text{obs}}(f)df},$$

where $y_{\text{obs}}(f)$ is the tissue-mean amplitude spectrum and $E(f)=\text{c}+a\cdot e^{-b\cdot f}$ is the fitted exponential background. This measure captures the relative energy excess of the Lorentzian component above the background within the second peak frequency band, which was evaluated for each of the tissue types (WM, GM, and CSF) separately.

## 3 Results

### 3.1 Salient second spectral peak in WM BOLD signals

**Figure 1:**
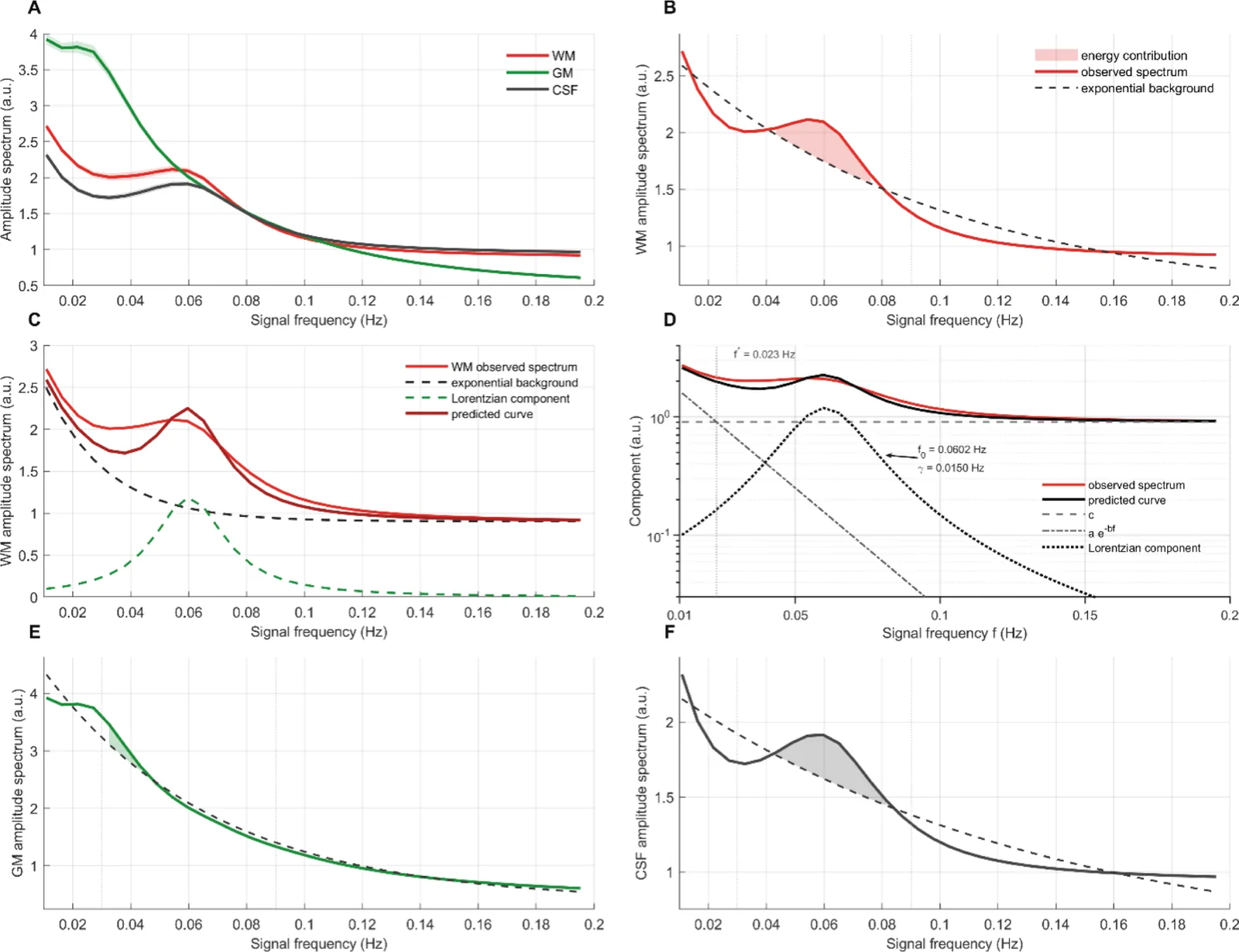
Tissue-specific low frequency spectra of BOLD signals and energy contributions from the second peak. (**A**) Group-mean spectra of WM, GM, and CSF show distinct tissue-specific spectral profiles. (**B**) In WM, the group-mean BOLD amplitude spectrum shows a local low frequency component above the fitted exponential background; the shaded area denotes its energy contribution within 0.03–0.09 Hz. (**C**) The WM spectrum is decomposed into an exponential background and Lorentzian component, the sum of which well predicts the observed spectrum. (**D**) Log-scale visualization of the fitted spectral components, highlighting the exponential background, constant floor, and Lorentzian contribution underlying the WM spectrum. (**E-F**) The same energy contribution analysis is performed for GM and CSF.

Figure 1 shows the group-mean amplitude spectra of WM, GM and CSF computed from the HCP young-adult cohort of 120 healthy participants. Across the three types of tissue examined, the spectra exhibited a grossly exponential decline trend with frequency (Fig. 1A). Compared to GM, however, the WM spectrum was noticeably elevated above the exponential background within the frequency range of ~ 0.03–0.09 Hz (Fig. 1B). The salient second peak in the spectrum could be well captured by a combined exponential-Lorentzian model, which yielded a remarkably good group-level fit (R^2^ = 0.9998) with a peak center frequency of ~ 0.06 Hz (Fig. 1C). Compared with the pure exponential model (R^2^ = 0.9274), the combined model substantially improved the fit and was strongly favored by AIC (ΔAIC = 171.98). The component-resolved representation further revealed a crossover between the exponential background and the frequency-independent floor at approximately 0.023 Hz (Fig. 1D). Note that the Lorentzian component reached its maximum at ~ 0.06 Hz, by which the exponential component had substantially decayed. Thus, the ~ 0.06 Hz elevation appears to be a localized spectral feature superimposed on the broadband background rather than a spreading of the low frequency components. These findings support the presence of a distinct second spectral component in WM BOLD signals, which was subsequently evaluated at the subject and voxel levels in this work.

Quantitative analyses showed that the second spectral component contributed an average level of 11.8% energy in WM signals within the frequency band of 0.03–0.09 Hz across the cohort of 120 subjects studied, which is significantly higher than that of GM signals (6.0%, Fig. 1E) based on paired t-test (p < 0.001). Interestingly, BOLD signals in CSF also exhibited comparable energy contributions from second spectral component (Fig. 1F) peaked ~ 0.06 Hz, suggesting that this peak could be driven by a common factor between WM and CSF.

### 3.2 Robust second spectral peak at the individual level

**Figure 2:**
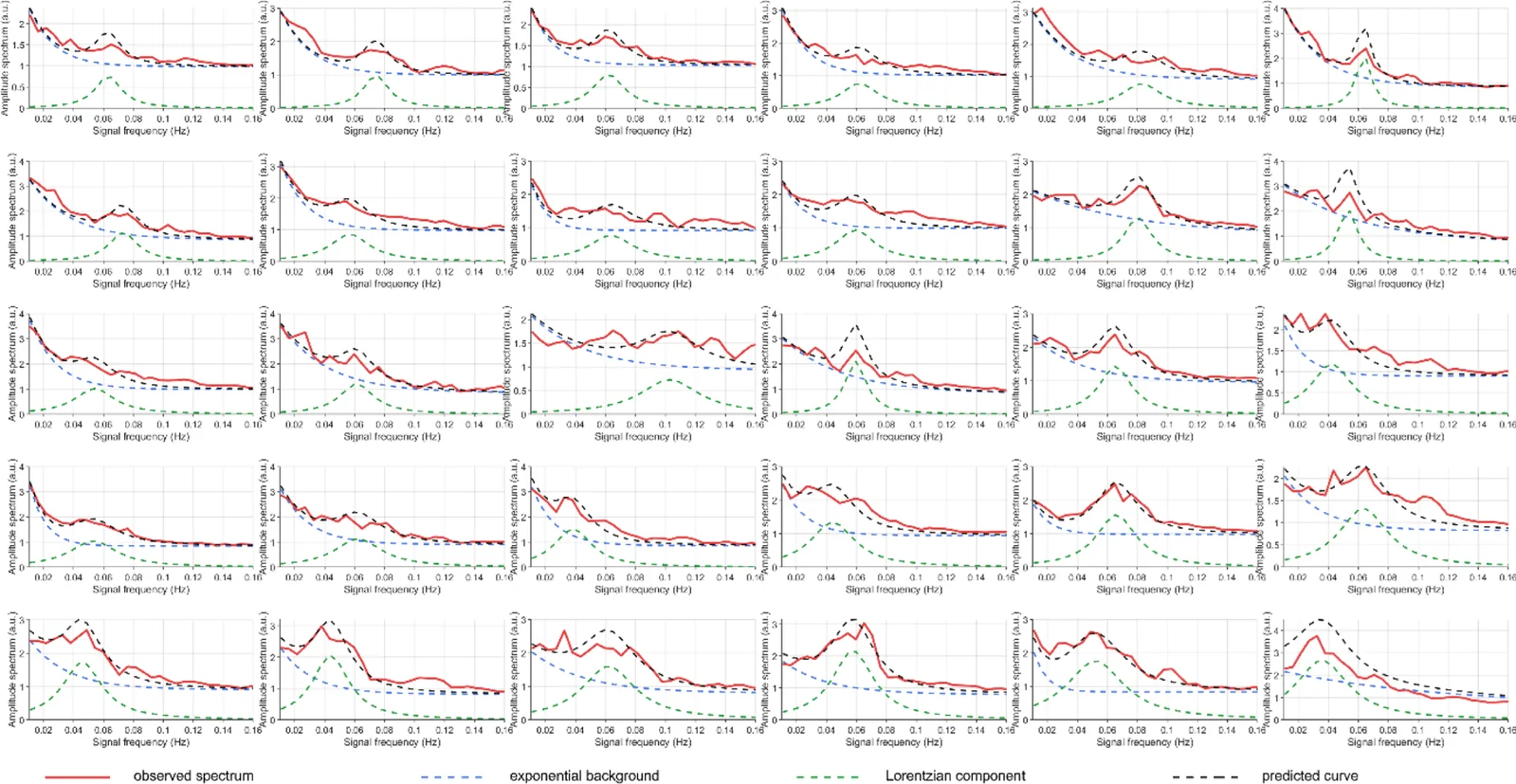
Mathematical modelling of amplitude spectra in WM BOLD signals from 30 randomly selected subjects. A combined exponential-Lorentzian model was used and the spectra were averaged across WM.

The WM second spectral peak was robustly observable in the 120 subjects studied, with the vast majority of the signal spectra well captured by a combined exponential-Lorentzian model. Figure 2 shows the mathematical modeling of WM signal spectra for 30 randomly selected subjects, and Table 1 summarizes model adequacy measure ΔAIC and model parameters including exponential background parameters (a, b and c) and second-peak parameters ($f_{0}$, peak height, width (γ), coherence timescale $\left({\text{τ}}_{\text{coh}}\right)$, and spectral amplitude contribution). As indicated by the large ΔAIC, the combined exponential-Lorentzian model overwhelmingly outperformed the pure exponential model, which illustrates the necessity of modelling the peak component in addition to the exponential decay. The peak center frequency tended to be concentrated around 0.06 Hz (i.e., smaller inter-subject variances), whereas peak height, width, energy contribution and coherence timescale had much larger variability across the subjects. The convergency and divergency in the model parameters together potentiate the second spectral peak in WM to be used an individual-level phenotype.

**Figure 3:**
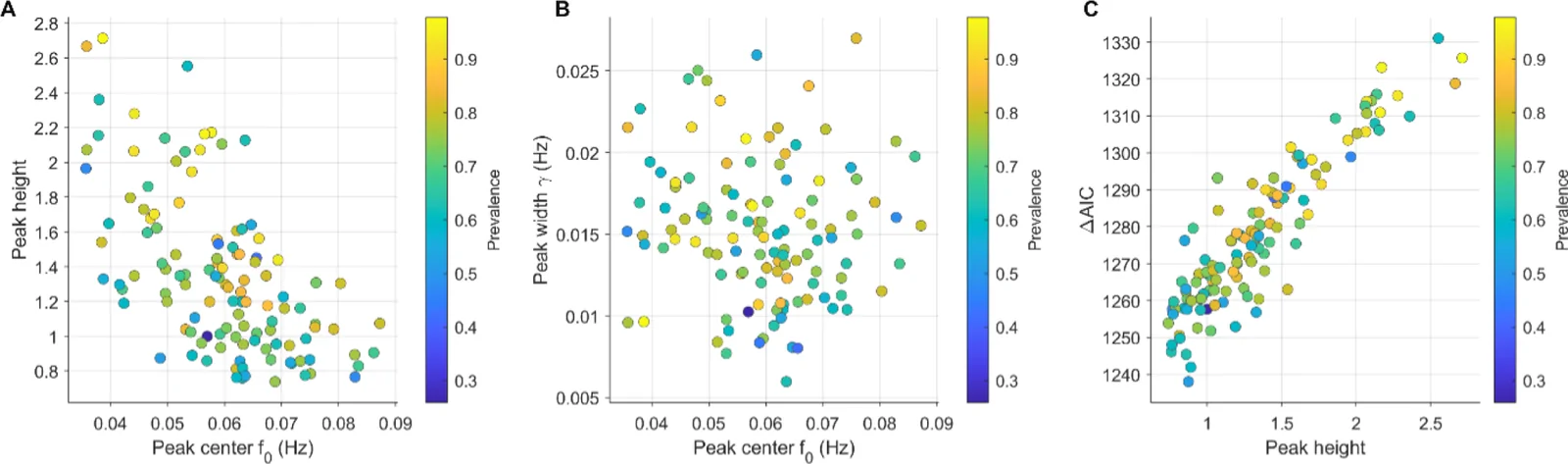
Relationships between model parameters. (**A**) Peak center frequency was negatively correlated with peak height. (**B**) Peak center frequency showed no clear correlation with peak width γ. (**C**) Peak height was positively associated with model improvement, measured by ΔAIC. Color denotes valid-peak prevalence across white matter.

We further examined the relationships between the model parameters, which revealed that the center frequency had a strong inverse relation with the peak height (r=−0.507, p < 0.001) (Fig. 3A), but had little relation with the peak width (r=−0.223) (Fig. 3B). This indicates that the process that drives the second spectral peak contributes to the signal spectra in a multiplicative manner (particularly around the center of the peak), which is typical of how physiological processes exert influences.

### 3.3 Structured spatial organization of the second peak within white matter

**Figure 4:**
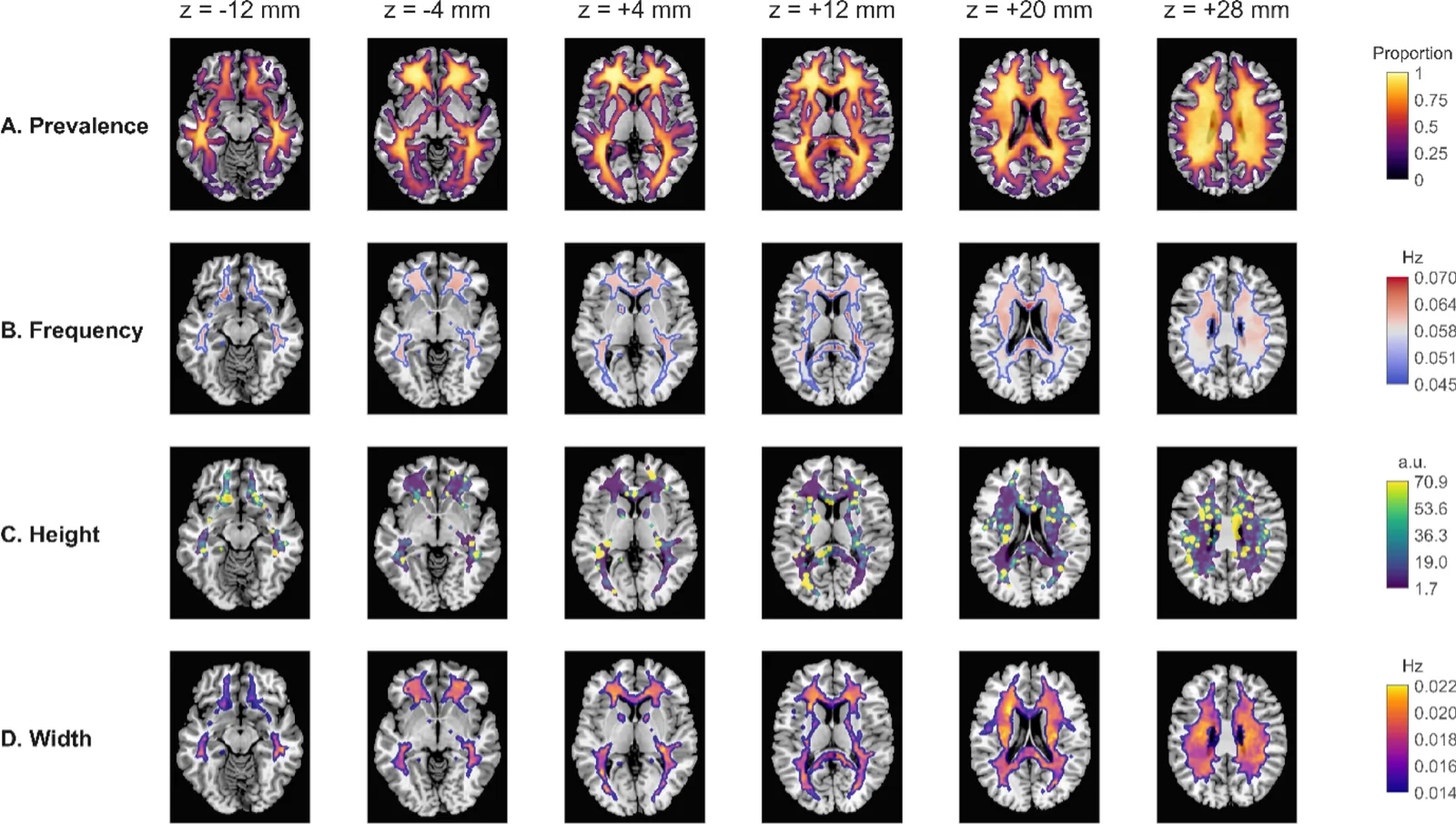
Spatial organization of the second spectral peak in white matter. Voxelwise maps show the spatial distribution of second-peak metrics within white matter. Rows respectively represent valid-peak prevalence (A), mean peak center frequency (B), mean peak height (C), and mean peak width (D) across subjects. Columns show axial slices from inferior to superior white matter. Valid-peak prevalence indicates the proportion of subjects with a stable second peak at each voxel.

Voxelwise analyses showed that the second spectral peak was detectable across most portions of WM in each subject, with a median fraction of 73.8% of WM voxels possessing a valid peak. The valid-peak prevalence across the 120 subjects studied exhibited clear spatial organization within WM, which was highest in deep WM regions anatomically corresponding to the corpus callosum, internal capsule, and corona radiata, and gradually decreasing toward peripheral WM regions (Fig. 4A). Peak center frequency, however, tended to have a gradient along the anterior-posterior direction particularly in the upper slices (Fig. 4B). This frequency gradient grossly agrees with that found in the cortex previously (Baria et al., 2011), which again attests to the notion that white matter contains neural activity information just like gray matter. By contrast, peak height and width appeared to be more uniformly distributed throughout the brain (Fig. 4C&D). Together, these findings show that the second peak has certain organization patterns that are aligned with macroscopic WM anatomy, suggesting that the peak was mediated by physiological processes.

### 3.4 Spectral evolutions with age: exploratory observations

**Figure 5:**
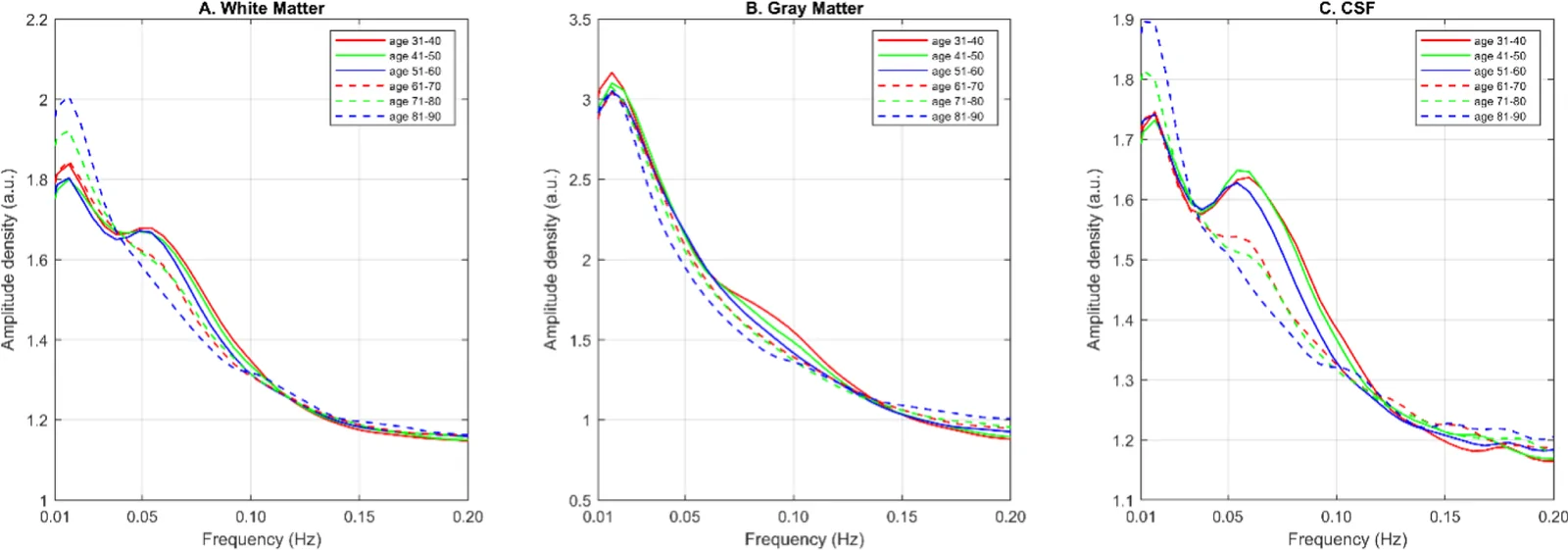
Mean amplitude spectra in six age groups of the HCP-Aging cohort. From left to right panels are amplitude spectra of WM, GM and CSF signals respectively, and the six age groups are 31–40, 41–50, 51–60, 61–70, 71–80, and 81–90 years.

We qualitatively compared amplitude spectra of BOLD signals in WM, GM, and CSF across six age groups, with results shown in Fig. 5. Evolutions of the second spectral peak with age can be clearly appreciated for WM. More specifically, the height of the second spectral peak centered around ~ 0.057 Hz decreased with age, with frequency range spanning from ~ 0.03 to ~ 0.10 Hz. Close inspections reveal that the three groups aged 31–60 years tended to have small spectral differences, and the same phenomenon was also observed for the two groups aged 61–80 years, but the subjects within this age range showed large decreases in the second peak from the subjects within the age range of 31–60 years. The group of subjects aged 81–90 years continued the decline trend in spectra, with the second spectral peak virtually vanished.

GM signals, on the other hand, did not have a pronounced second spectral peak in any of the age groups, although there are some subtle spectral perturbations around ~ 0.08 Hz. Meanwhile, much like the spectral evolution in WM, CSF signals in the HCP-Aging cohort also exhibited a prominent aging-driven spectral evolution, most notably progressive decreases of the height of the second spectral peak with age. This again attests to the notion that the second spectral peak in WM and CSF signals might share the same physiology.

## 4 Discussion

This study mathematically models and quantitatively characterizes a robustly observable second peak in the spectra of white matter BOLD signals. This spectral signature was conspicuously prominent in group-averaged spectra and ubiquitously present in individual subjects, demonstrating reliable existence of this spectral phenomenon. Distributions of the second spectral peak were found not to be uniform throughout white matter; rather, they exhibited spatial gradients that grossly followed large scale white matter anatomy, with greater manifestations in deep major tracts than subcortical regions. Analyses of the HCP-Aging cohort further revealed that this spectral signature evolves with age, with a progressively subdued peak as the age increases. The second spectral peak was also clearly visible in BOLD signals of the CSF in the lateral ventricles, which tended to suggest that cross-tissue physiological processes might mediate such a signal phenomenon. Taken together, these findings support the notion that the WM second peak is a genuine, robust and physiologically meaningful spectral feature.

Physiological interpretations of the second spectral peak, however, are not readily derivable from the functional MRI data analyzed in this study. Distribution patterns of the second peak in white matter, and especially its concomitant presence in CSF, appear not to be directly consequential to conventional regimes of neural conductions, which in principle proceed along the course of white matter fibers. Instead, it is more plausible that it might reflect a low frequency fluid-related dynamics shared between white matter and CSF. Recently, Fultz et al. (2019) discovered a similar spectral peak in CSF signals, which was more pronounced during human sleep when the brain tissue presumably clears wastes through glymphatic circulations. It was also observed that CSF inflow tended to be preceded by hemodynamic BOLD increase (Fultz et al., 2019). To account for the temporal ordering, Kerskens et al. (2026) proposed a causal chain of vascular expansion and fluid displacement, which is modulated by a mechanical filtering process that allows enhanced dynamics of waster clearance near ~ 0.05 Hz. In recognition of these studies, the vascular and glymphatic dynamics, which are intimately coupled (Prasuhn et al., 2024), deserve particular attention as a candidate explainer for the second peak observed in our study. This is indeed supported by several key and converging observations we made: the frequency range of the second spectral peak, the presence of concurrent phenomenon in CSF, and the strong preference of the spectral peak for deep white matter regions that are spatially proximal to the CSF-containing lateral ventricles. Nevertheless, the findings from the current study do not lend themselves to such a mechanism, but could be evocative of a potential direction for further investigations.

From the practical perspective, the white matter second peak may be regarded as a simple yet valuable spectral phenotype. This phenotype could be used as a novel biomarker for brain disorders or deficits, as evidenced by our exploratory analysis of aging populations in this work. Current functional neuroimaging biomarkers are almost exclusively derived from gray matter (Horwitz et al., 2011). Exploitations of BOLD signal spectra in white matter may provide a new noninvasive window into low frequency physiological dynamics in human brain. In this sense, white matter may serve not merely as a passive “background” tissue that silently supports brain function, but as a particularly informative readout of physiological processes that are otherwise unavailable from conventional gray matter centered analyses (see more discussions below).

It should be noted that, in drastic contrast to white matter, gray matter did not exhibit a clear second spectral peak in its resting-state BOLD signals. A possibility for the dichotomy is that ∼0.05Hz global fluctuations in brain activities emerge primarily during slow wave sleep, which are substantially reduced during wakefulness (Kerskens et al., 2026). This cognitive state dependency is highly pronounced for gray matter, in which rapid perceptual, cognitive and sensorimotor updating is repeatedly performed that overwhelms the globally fluctuating signals. White matter, on the other hand, has much reduced vascular density (Nonaka et al., 2003; Jensen et al., 2006), which translates to weak BOLD signals that arise from transmitting cortical activities such that the ~ 0.05Hz global fluctuations are still relatively prominent even during wakefulness. It is emphasized that this explanation, along with the aforementioned mechanistic interpretation for white matter — particularly those involving vascular-glymphatic circulations — remain speculative at this stage. Further work combining higher temporal resolution fMRI, physiological recordings, and structural imaging of vascular-glymphatic circulations is warranted for clarifying specific biological processes underlying this phenomenon.

## Figures and Tables

**Table 1:** Subject-level parameters of the white matter second spectral peak

Metric	Mean	SD	Median	IQR
ΔAIC, pure exponential minus exponential-Lorentzian model	1065.4583	109.5432	1122.9730	148.9222
Exponential amplitude (a) (a.u.)	2.9398	1.1427	2.6496	1.3502
Exponential decay constant (b) (Hz^−1^)	51.366	25.581	47.197	31.249
Constant floor (c) (a.u.)	0.8927	0.1133	0.9039	0.1517
Lorentzian area (A) (a.u.)	0.0644	0.0296	0.0561	0.0364
Peak center frequency $f_{0}$ (Hz)	0.0597	0.0123	0.0602	0.0154
Peak height (a.u.)	1.3457	0.4569	1.2891	0.5774
Peak width γ (Hz)	0.0153	0.0044	0.0150	0.0055
Coherence timescale ($\left({\text{τ}}_{\text{coh}}\right)$) (s)	11.326	3.556	10.605	3.979
Energy contribution (%)	11.8120	3.4125	11.3116	4.6163
